# Genotypic and Phenotypic Characteristics of 29 Patients With Rare Types of Osteogenesis Imperfecta: Average 5 Years of Follow-Up

**DOI:** 10.3389/fgene.2021.622078

**Published:** 2021-07-16

**Authors:** Lei Xi, Hao Zhang, Zhen-Lin Zhang

**Affiliations:** Shanghai Clinical Research Center of Bone Disease, Department of Osteoporosis and Bone Disease, Shanghai Jiao Tong University Affiliated Sixth People’s Hospital, Shanghai, China

**Keywords:** osteogenesis imperfecta, follow-up, rare type, bisphosphonates, bone mineral density

## Abstract

Osteogenesis imperfecta (OI) is a rare genetic disorder characterized by bone fragility and abnormal connective tissue. Ninety percent of OI patients are caused by two mutations of *COL1A1* and *COL1A2*, and more investigation was needed to better understand the rare types of OI. We followed up 29 patients with rare types of OI for an average of 5.4 years, and genotype, height, bone mineral density (BMD), blood biochemical indexes, misdiagnosis, and fracture were recorded. *IFITM5* gene mutation was found in 18 patients (62.1%), which represents the most common pathogenic gene of rare types of OI in Chinese population. Thirteen cases had once been misdiagnosed, and the initial misdiagnosis rate was 44.8% (13/29). The higher misdiagnosis rate should be paid attention to by clinicians and healthcare providers, and we also give corresponding suggestions. Compared with the non-bisphosphonate treatment group, patients treated with bisphosphonates had higher lumbar spine BMD, fewer fractures, and lower levels of β-CTX and osteocalcin. However, there was no significant difference between OI type V patients and non-type V patients. Our study enriched the knowledge of genotype and phenotype characteristics of OI patients with rare types and bisphosphonate therapy.

## Introduction

Osteogenesis imperfecta (OI) is a rare genetic disorder characterized by bone fragility and abnormal connective tissue. The population prevalence of OI is about 1/15,000–1/20,000 (Stoll et al., 1989; Forlino et al., 2011). Common clinical manifestations include repeated fractures, blue sclera, dentinogenesis imperfecta, and hearing impairment.

Sillence and Rimoin (1978) classified OI into four types (type I–IV) based on the severity of the disease. Additional OI types (type V and higher) have more recently been identified, based on genetic findings (Mortier et al., 2019). So far, more than 20 different genes have been found to be related to the pathogenesis of OI. Ninety percent of OI patients are caused by two mutations of *COL1A1* (MIM 120150) and *COL1A2* (MIM 120160) encoding α1 chain and α2 chain of type 1 collagen (Orioli et al., 1986; Sykes et al., 1986; Primorac et al., 2001; Marini et al., 2017; Moosa et al., 2019). The phenotypes of these two mutation genes are basically within the four types described by Sillence. However, the OI phenotype caused by rare pathogenic genes that are related to collagen synthesis, posttranscriptional modification and secretion, osteoblast differentiation, etc, is often more special, and skeletal deformities are also more serious (Rauch and Glorieux, 2004). For example, OI type V caused by the [Interferon-induced transmembrane protein 5(*IFITM5*); MIM 614757] gene mutation will be accompanied by the formation of huge callus, calcification of interosseous membrane, and subluxation of radial capitulum (Semler et al., 2012); OI type XI caused by mutations of [FK506 binding protein 10 (*FKBP10*); MIM 607063] is characterized by congenital joint contractures (Zhou et al., 2014); patients with the *P4HB* (Prolyl 4-hydroxylase; MIM: 176790) mutation may present with craniosynostosis, exophthalmos, blepharoptosis, and even hydrocephalus (Rauch et al., 2015). Approximately 90% of the 3,000 individuals whose mutations have been included in the OI variant database^1^ have alterations in either *COL1A1* or *COL1A2*. In view of the above situation, we defined the OI patients with *COL1A1* and *COL1A2* gene mutations as common mutations, and the patients with OI with other gene mutations as rare mutations.

No cure exists for OI. For patients with different conditions and ages, the treatment objectives and methods are different, mainly including drug treatment, orthopedic surgery, and physical rehabilitation. Recent studies have shown that bisphosphonates (BPS), Denosumab (a *RANKL* antibody), and sclerosin (*SOST*) are expected to increase bone density, improve bone microstructure, and reduce the risk of fractures in OI patients. BPS are currently used in the therapy of OI to increase bone mineral density (BMD) observed in OI patients caused by collagen type I mutations. However, different scholars have different opinions on the efficacy of bisphosphonates in the treatment of OI patients with rare types. Ranganath et al. (2016) reported that BPS may exacerbate callus hyperplasia and may therefore have to be used with caution in patients with OI type V; Trejo et al. (2017) concluded that intravenous bisphosphonate treatment during growth was beneficial in OI type VI, including an increase in lumbar spine areal BMD, a higher final height and some reshaping of vertebral bodies; Land et al. (2007) concluded that intravenous pamidronate was less effective in the treatment of OI type VI compared with OI caused by mutations in *COL1A1* or *COL1A2*; Keupp et al. (2013) did not find that bisphosphonates had a substantial effect on OI patients due to *WNT1* mutations.

In this study, we evaluated the mid-term clinical course of patients with rare types of OI (Zhang et al., 2012, 2013, 2017, 2018; Cao et al., 2019a,b). The height, BMD, and fracture frequency of patients with or without bisphosphonates were compared. The misdiagnosis of OI patients was analyzed and relevant suggestions were put forward.

## Materials and Methods

### Subjects

The study population is composed of the 29 rare types of OI patients who were assessed at the Shanghai Jiao Tong University Affiliated Sixth People’s Hospital between May 2005 and September 2020. Gene mutations in all patients were previously published in journals (Zhang et al., 2012, 2013, 2017, 2018; Cao et al., 2019a,b). In addition, 185 Chinese probands of OI who carried mutations in the *COL1A1* or *COL1A2* genes have been reported in our studies (Xi et al., 2021). The study was approved by the Ethics Committee of the Shanghai Jiao Tong University Affiliated Sixth People’s Hospital. Relevant clinical data and laboratory data were gathered by retrospective chart review and parental interviews.

### Clinical Evaluation

Detailed clinical features were collected through a medical examination. Medical history was collected based on patient’s files and information obtained from the patient or their parents. Height was measured using a wall-mounted stadiometer and recorded to the nearest 0.1 cm: for infants and children unable to stand, use the length of supine position instead; if the legs are unequal, use the longer legs as the measurement basis. BMD was measured by Dual-energy X-ray absorptiometry (DXA) (GE Lunar Corp., Madison, WI, United States), and the measurement sites were lumbar spine (LS, L1–L4) and left femoral neck (FN). The results were transformed to age- and sex-specific *Z*-scores combining 0- to 18-year-old children and adolescents in China (Tan et al., 2007; Li et al., 2009; Xu et al., 2013).

Information on bone fractures was confirmed by medical record or bone X-ray images. Vertebral compression fracture (VCF) was judged by Genant semi-quantitative method (Genant et al., 1993). Spine radiographs were used to determine if there was scoliosis. Scoliosis was diagnosed when a Cobb angle of ≥10° was observed (Sato et al., 2016).

The exome of probands, their parents, and siblings were sequenced to identify the pathogenic gene. As reported in our previous studies (Zhang et al., 2012, 2013, 2017, 2018; Cao et al., 2019a,b), we designed a targeted gene sequencing panel for next-generation sequencing to economically and conveniently establish a comprehensive molecular diagnostic method for rare type OI and confirmed the possible mutations using Sanger sequencing.

### Treatments

The bisphosphonates used in the research include oral alendronate, 70 mg regular weekly (Merck & Co., Inc.) and intravenous ibandronate, 2 mg regular 3 monthly (Biomedical Engineering Center of Hebei Medical University). Among them, ibandronate was the main treatment in our center before 2010, and alendronate has been the main treatment since 2010. In addition, the patients were supplemented with 600 mg calcium and 200 IU vitamin D3 daily (Caltrate, Pfizer).

### Statistical Analyses

All data were processed by SPSS 24.0 (SPSS Inc., Chicago, IL, United States). Comparison between two or more groups: for measurement data, Student’s *t*-test is used for normal distribution; Wilcoxon rank sum test is used for non-normal distribution; chi-square test is used for counting data. *p*-values <0.05 were considered significant.

## Results

### Patient Characteristics

Twenty-nine patients (55.2% male) were followed for an average of 5.4 years. Eighteen individuals (62.1%) with OI had *IFITM5* gene mutations; *LEPRE1*, *SEC24D*, and *SERPINF1* mutations were found in two cases (6.8, 6.8, and 6.8%, respectively). Other mutated genes included *P4HB*, *PLS3*, *TMEM38B*, *WNT1*, and *FKBP10* (3.5, 3.5, 3.5, 3.5, and 3.5%, respectively), as previously described (Zhang et al., 2012, 2013, 2017, 2018; Cao et al., 2019a,b).

All patients were born with normal height and weight and came from non-consanguineous families except patient C23. At birth, no fractures or skeletal abnormalities were found. Twenty-six patients (89.7%) experienced their first fracture before puberty, followed by a large number of fractures, and three patients (10.3%) had no fracture (Table 1). All patients had no hearing loss. One patient had blue sclera and three patients had dentinogenesis imperfecta. Nine individuals presented with late fontanel closure (>2 years). Mobility impairments were present in 13 patients (Table 2).

**TABLE 1:** Demographic features and fracture incidence of rare types OI patients.

**No.**	**Sex**	**Age of first visit (years)**	**Mutant gene**	**Mutation site**	**Classical Sillence classification**	**Age of the first fracture (years)**	**Number of fractures**	**Bisphos phonate**	**Time of follow-up**
C1	F	45	*P4HB*	c.1198T > C (p.C400R)	III	2	20	Yes	9
C2	M	11	*PLS3*	c.892-1G > A	IV	4	5	Yes	10
C3	F	17	*TMEM38B*	c.150C > G + c.507G > A (p.W169X + p.S50R)	III	1	26		6
C4	M	10	*WNT1*	c.500dupG + c.506G > A (p.C170Lfs + p.G169D)	IV	6	8	Yes	4
C5	F	17	*LEPRE1*	c.1675dupC + c.1473 + 3A > C (p.Leu559ProfsX24 + IVS9 + 3A > C)	I	2	4		6
C6	M	24	*LEPRE1*	c.1466T > C + c.1915-1G > A (p.Leu489Pro + IVS13-1G > A)	I	2	5		6
C7	M	23	*SEC24D*	c.2723G > A + c.2842T > C (p.Cys908Tyr + p.Ser948Pro)	IV	1	11		4
C8	M	7	*SEC24D*	c.938G > A + c.875C > T (p.Arg313His + p.Pro292Leu)	IV	1.5	9		5
C9	F	11	*SERPINF1*	c.1067T > A (V356E)	IV	2	8		4
C10	M	4	*SERPINF1*	c.283 + 473_643 + 104del (p.Ala96_Gly215del)	I	3	4		5
C11	M	12	*FKBP10*	c.813_814delGA + c.831delC (p.Glu271AspfsX101 + p.Gly278AlafsX20)	IV	10	9	Yes	5
C12	F	39	*IFITM5*	c.-14C > T	I	8	6		4
C13	M	16	*IFITM5*	c.-14C > T	I	4	7	Yes	4
C14	M	17	*IFITM5*	c.-14C > T	I	3	7		3
C15	M	1	*IFITM5*	c.-14C > T	III	0.7	4		3
C16	M	64	*IFITM5*	c.-14C > T	I	0	0		5
C17	M	39	*IFITM5*	c.-14C > T	I	0	0		5
C18	F	38	*IFITM5*	c.-14C > T	I	0.7	17		5
C19	F	29	*IFITM5*	c.-14C > T	I	0.2	18		5
C20	F	10	*IFITM5*	c.-14C > T	IV	1.5	9	Yes	5
C21	M	48	*IFITM5*	c.-14C > T	III	3	15		8
C22	F	14	*IFITM5*	c.-14C > T	IV	2	8		8
C23	F	25	*IFITM5*	c.-14C > T	IV	5	10	Yes	3
C24	F	5	*IFITM5*	c.-14C > T	I	0	0		3
C25	F	29	*IFITM5*	c.-14C > T	III	4	13	Yes	8
C26	M	7	*IFITM5*	c.-14C > T	IV	3	7	Yes	11
C27	F	18	*IFITM5*	c.-14C > T	I	4	8		6
C28	M	4	*IFITM5*	c.-14C > T	IV	1.5	6		4
C29	F	14	*IFITM5*	c.-14C > T	III	1.2	11	Yes	4

**TABLE 2:** Summary of clinical, radiological features, and comorbidity with rare types of OI.

	**Patients with different mutated genes**								
	***IFITM5***	***P4HB***	***PLS3***	***TMEM38B***	***WNT1***	***LEPRE1***	***SEC24D***	***SERPINF1***	***FKBP10***
Male/Female	8/10	0/1	1/0	0/1	1/0	1/1	2/0	1/1	1/0
**Clinical features**									
Blue sclera	(−)	(−)	(−)	(−)	(−)	(−)	(−)	(±)	(−)
Dentinogenesis imperfecta	(±) 1/18	(−)	(−)	(−)	(−)	(−)	(+)	(−)	(−)
Hearing loss	(−)	(−)	(−)	(−)	(−)	(−)	(−)	(−)	(−)
Joint hyperextension	(±) 1/18	(−)	(+)	(−)	(+)	(−)	(−)	(−)	(−)
Late fontanel closure (>2 years)	(±) 5/18	(+)	(−)	(+)	(−)	(−)	(+)	(−)	(−)
Mobility impairment	(±) 7/18	(+)	(−)	(+)	(−)	(+)	(±)	(−)	(+)
**Radiological characteristic**									
Scoliosis	(±) 7/18	(+)	(−)	(+)	(+)	(−)	(±)	(±)	(+)
Vertebral compression fracture	(±) 7/18	(−)	(−)	(−)	(+)	(−)	(±)	(+)	(+)
Femoral fracture	(±) 9/18	(+)	(−)	(−)	(+)	(+)	(+)	(+)	(+)
**Comorbidity**									
Mitral and tricuspid regurgitation	(−)	(−)	(−)	(−)	(+)	(−)	(−)	(−)	(−)
Inflammation of hips	(±) 5/18	(−)	(−)	(−)	(−)	(−)	(−)	(−)	(−)
Impaired vision	(−)	(+)	(−)	(−)	(−)	(−)	(−)	(−)	(−)
Intraventricular block	(±) 1/18	(−)	(−)	(−)	(−)	(−)	(−)	(−)	(−)
Cholelithiasis	(±) 1/18	(−)	(−)	(−)	(−)	(−)	(−)	(−)	(−)

### The Misdiagnosis Condition in Rare Types of OI

**FIGURE 1:**
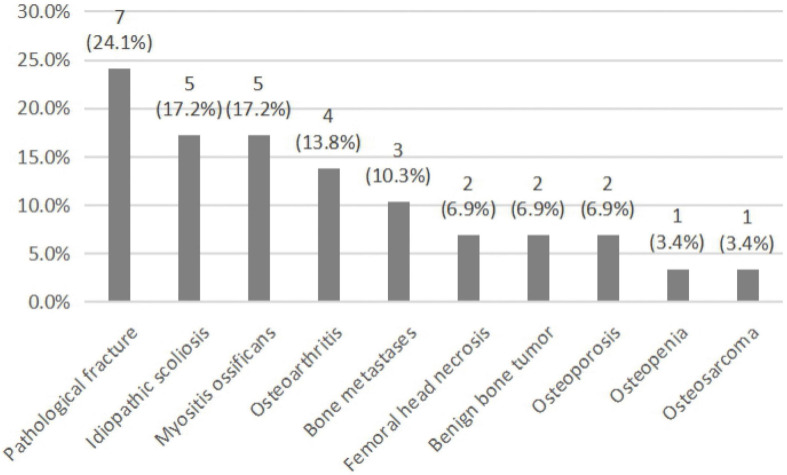
The misdiagnosis condition in rare types of OI.

Among the 29 rare types of OI patients, 13 cases had once been misdiagnosed and the initial misdiagnosis rate was 44.8% (13/29). There were 32 case-times of misdiagnoses that occurred among these patients. As is shown in Figure 1, rare types of OI patients were often misdiagnosed as pathological fracture, idiopathic scoliosis, and myositis ossificans, followed by osteoarthritis, bone metastases, femoral head necrosis, benign bone tumor, osteoporosis, osteopenia, and osteosarcoma.

### Medical Treatment

Ten patients received treatment of bisphosphonates, starting between 6 and 45 years of age (median, 12 years). The total duration of bisphosphonate treatment was between 1 and 5 years (median, 3.2 years). Among the 10 patients, six patients had *IFITM5* gene mutation, and the other four patients had *P4HB*, *PLS3*, *WNT1*, and *FKBP10* gene mutation, respectively. The patient with *PLS3* gene mutation had been treated with ibandronate for 2 years and alendronate for 3 years; the patient with *FKBP10* gene mutation had been treated with ibandronate for 1 year and alendronate for 2 years, and the other patients were treated with only alendronate. Height and BMD of the lumbar spine were converted to age- and sex-specific *Z*-scores. Compared with the non-bisphosphonate treatment group, patients treated with bisphosphonates had higher lumbar spine BMD (0.54, increase of lumbar spine BMD *Z*-score per year, *p* < 0.001 for adult), fewer fractures (0.7 and 0.5, frequency of fractures per year, *p* = 0.042 for adult and *p* < 0.001 for children), lower level of beta cross-linked carboxy-terminal telopeptide of type I collagen (β-CTX) (348.1 ± 60.4 and 154.5 ± 37.2, both *p* < 0.001), and lower level of osteocalcin (OC) (26.1 ± 12.2 and 20.2 ± 4.7, both *p* < 0.001). However, there was no significant difference in *Z*-value of height between the group with bisphosphonate treatment and the group without bisphosphonate treatment (−0.367 and 0, increase of height *Z*-score per year, *p* = 0.867 for children and *p* = 0.503 for adult) (Table 3).

**TABLE 3:** The long-term clinical course of rare types of OI patients.

	**Group with bisphosphonate treatment**		**Group without bisphosphonate treatment**		**Type V patients**		**Non-type V patients**		***P-*value**			
	**Children**	**Adult**	**Children**	**Adult**	**Children**	**Adult**	**Children**	**Adult**				
Age^a^	13.6 ± 2.9	31.6 ± 12.3	11.0 ± 5.2	25.6 ± 7.2	10.4 ± 4.5	27.8 ± 7.2	13.8 ± 4.0	29.8 ± 14.0				
Frequency of fractures (per year)	0.5 (0.2, 0.9)	0.7 (0, 1)	2.0 (1.6, 2.7)	1.5 (0.9, 1.6)	1.5 (0.8, 2.3)	1 (0.7, 1.6)	1.5 (0.7, 2.2)	1.3 (0.9, 1.6)	<0.001^b^	0.042^c^	0.901^d^	0.644^e^
Increase of height *Z*-score (per year)	−0.367 (−0.534, 0.190)	0 (−0.162, 0)	−0.167 (−0.433, 0.044)	−0.025 (−0.054, 0)	−0.157 (−0.363, 0)	−0.072 (−0.002, 0.016)	−0.12 (−0.267, 0.072)	−0.013 (−0.033, 0)	0.867^b^	0.503^c^	0.801^d^	0.489^e^
Increase of Lumbar spine BMD *Z*-score (per year)	0.54 (0.433, 0.733)	0.04 (−0.233, 0.067)	−0.017 (−0.079, 0.05)	0 (0, 0.104)	0.31 (−0.025, 0.53)	0 (−0.002, 0.012)	0.24 (0, 0.417)	0.022 (0, 0.025)	<0.001^b^	0.355^c^	0.782^d^	0.301^e^
ALP (U/L)^a^	282.2 ± 79.6	63.4 ± 17.9	634.6 ± 195.5	99.4 ± 35.0	625.8 ± 306.1	157.0 ± 93.7	352.6 ± 212.4	142.7 ± 57.7	<0.001^b^	0.070^c^	0.099^d^	0.876^e^
β-CTX (ng/L)^a^	348.1 ± 60.4	154.5 ± 37.2	683.1 ± 159.0	322.1 ± 61.9	367.4 ± 358.2	106.6 ± 94.8	691.5 ± 390.0	417.8 ± 291.7	<0.001^b^	<0.001^c^	0.272^d^	0.152^e^
OC (ng/ml)^a^	26.1 ± 12.2	20.2 ± 4.7	79.0 ± 16.1	39.8 ± 10.9	34.9 ± 12.2	26.3 ± 9.7	72.4 ± 26.3	26.1 ± 9.4	<0.001^b^	<0.001^c^	0.054^d^	0.594^e^

*^*a*^Data were obtained at the time of the last follow-up visit. ^*b*^Children with bisphosphonate treatment vs. children without bisphosphonate treatment. ^*c*^Adult with bisphosphonate treatment vs. adult without bisphosphonate treatment. ^*d*^Children of OI type V patients vs. children of non-type V patients. ^*e*^Adult of OI type V patients vs. adult of non-type V patients; BMD, bone mineral density; ALP, alkaline phosphatase; β-CTX, beta cross-linked carboxy-terminal telopeptide of type I collagen; OC, osteocalcin.*

### Longitudinal Growth and Bone Densitometry

**FIGURE 2:**
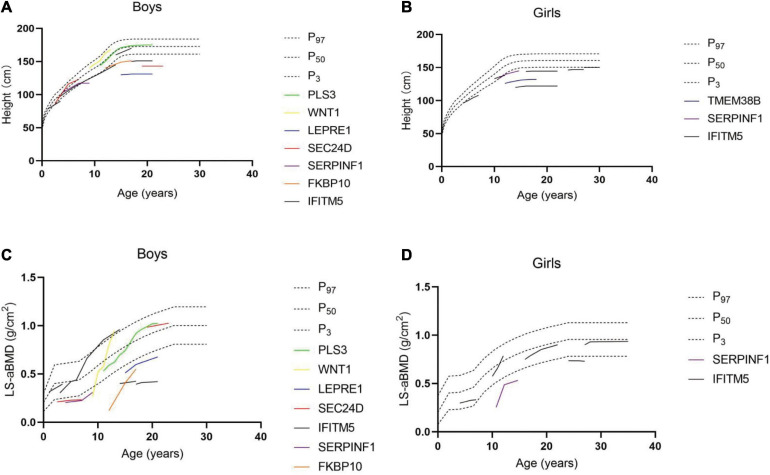
Follow-up results of height in male **(A)** and female **(B)** patients. Follow-up results of bone mineral density of lumbar spine in male **(C)** and female **(D)** patients.

Among the 29 rare types of OI patients, height follow-up records were available for 20 cases. The height was followed up between 2 and 11 years (median 5 years). Among the 20 patients, 12 patients reached the final height, of which 10 cases were lower than the standard height by two standard deviations (the lowest *Z*-score was −7.5), namely, five patients with *IFITM5* gene mutation, two patients with *LEPRE1* gene mutation, one patient with *FKBP10* gene mutation, one patient with *P4HB* gene mutation, and one patient with *SEC24D* gene mutation, and the heights of two patients were in the normal range (−0.5 and 0.4, *Z*-score), namely, one patient with *PLS3* gene mutation and one patient with *IFITM5* gene mutation. In addition, longitudinal growth curves and lumbar spine areal BMD curves of rare types of OI patients were drawn in the study. When plotted on growth curves, 67% of males and 100% of females were below the third percentile for height (Figures 2A,B). In children with OI, the peak of growth and development in adolescence was not obvious and the growth velocity was obviously behind that of children of the same age. One patient with *TMEM38B* gene mutation and one patient with *IFITM5* gene mutation appeared to have stopped growing at the age of 15, reaching the final height. After bisphosphonate treatment, the height of compressed vertebral body was restored partly, which was also reflected in height. In order to determine the effect of bisphosphonates on the height of patients, we divided the patients into four groups: children and adults treated with bisphosphonates, and children and adults without bisphosphonates treatment. The height of the patients in each group was matched with healthy people of the same race, age, and sex. We compared the annual increase in height *Z*-score of patients and found that bisphosphonates did not significantly improve short stature (*p* = 0.867 for children and *p* = 0.503 for adult). It should be noted that the patient with the *WNT1* gene mutation used bisphosphonates for 4 years and grew from 140 cm (−0.02, *Z*-score) to 167.8 cm (0.28, *Z*-score). Due to the serious scoliosis, it is impossible to measure the lumbar BMD in some patients, and a total of 18 patients were followed up. When plotted on curves, nine patients were below the third percentile for the BMD of lumbar spine (Figures 2C,D). Even if vertebral compression fracture and scoliosis can increase the measurements of lumbar BMD to a certain extent, lumbar BMD of most OI patients was still lower than that of normal people. By comparing lumbar BMD of the above four groups, bisphosphonates can increase BMD. Among them, compared with different groups of children (0.54 vs. −0.017, increase of lumbar spine BMD *Z*-score per year, *p* < 0.001), we believe that the earlier the use of bisphosphonates, the more obvious the increase of lumbar BMD. However, scoliosis continued to progress in some patients after bisphosphonate treatment. In addition, in the follow-up process, we found that some patients suffered from other diseases: one patient with *WNT1* gene mutation (C4) was confirmed mitral and tricuspid regurgitation; five patients with *IFITM5* gene mutation (C17–20 and C24) were confirmed to have hip inflammation; one patient with *P4HB* gene mutation (C1) showed impaired vision; electrocardiographic examination of the patient with *IFITM5* gene mutation (C14) revealed intraventricular block; cholelithiasis was found in the patient with *IFITM5* gene mutation (C23) by B-ultrasound.

### Fractures

Skeletal deformities were a common clinical manifestation of OI. In this study, 44.8 and 89.7% of the patients had scoliosis and long bone deformity of extremities, respectively. During the follow-up, there were 12 patients with vertebral compression fractures, namely, four patients with mild compression and eight patients with severe compression; nine patients had more than two compressed pyramids, and the most commonly affected vertebrae were T12 and L1. Eighteen patients had femoral fracture, 11 of which were unilateral and 7 were bilateral. During the follow-up, these patients underwent intramedullary rodding procedures with an average of 3.4 times (range, 1–10 procedures). Eighteen patients had rodding surgery (non-telescopic rod in 10 cases, telescopic rods in eight cases) at the lower extremities. The patient with the *IFITM5* gene mutation (C29) was followed up for 8 years and hyperplastic callus was significantly smaller than before, but it still existed. Some patients, such as the patient with *WNT1* gene mutation (C4) recovered the height of vertebral body to some extent after using bisphosphonate.

## Discussion

In this study, we diagnosed and followed up 29 patients with rare types of OI in 22 families with an average follow-up of 5 years. Among 29 OI patients, 18 cases were OI type V, which suggested that OI type V was more common in rare types of OI in China. Compared with patients with typical type I collagen gene mutation in our previous studies (Zhang et al., 2016), patients with rare types of OI had had lower BMD at the lumbar spine (*Z*-score, −2.3 ± 0.8, *p* = 0.016). Although there is no significant difference between patients with typical type I collagen gene mutation and patients with rare types of OI in fracture frequency, patients with rare types of OI generally had severe deformity of limbs and spine, which led to difficulty in daily activities, and typical extraskeletal manifestations, such as blue sclera (3.8 vs. 80.3%, *p* < 0.001) and dentinogenesis imperfecta (10.3% vs. 32.8%, *p* = 0.007), were less.

It should be emphasized that not all patients with OI type V have typical clinical manifestations such as hypertrophic callus, radial-head dislocation, and ossification of the interosseous membranes. Among 18 patients with OI type V, 15 had typical ossification of the interosseous membranes, radial-head dislocation, and 10 had typical hyperplastic callus. Interestingly, ossification of the interosseous membranes and radial-head dislocation are often accompanied, and the calcification only decreased and did not disappear during follow-up. In patients using bisphosphonates, the aggravation of callus has not been observed, which was in contrast to the 16-year-old boy reported by Ranganath et al. (2016). In addition, although patients with OI type V have the same mutation site of *IFITM5* gene, there are great differences in clinical phenotype. In the family that included patients C16–C20, patients C16 and 17 have not had fracture since follow-up, while patients C18, 19, and 20 have severe bone deformity and cannot walk. Similar to the study of Garbes et al. (2015) and Li et al. (2020), patients with the *SEC24D* gene mutation in this study have late fontanel closure, but no typical hydrocephalus. In fact, at present, the patient with *SEC24D* gene (C7) is 27 years old, and fontanel closure is still not closed. Considering that there are significant regional differences between Chinese and Caucasian populations, this may be due to different genetic backgrounds. In addition, whether the heterogeneity of clinical phenotype is related to other genetic factors, modifying genes and so on needs further study.

Bisphosphonates are widely used in the treatment of OI patients (Trejo et al., 2017). In order to explore the effect of bisphosphonates on BMD, height, and fracture in patients with rare type OI, this study compared the follow-up data between different groups of patients. Previous studies have shown that fracture frequency and BMD of OI patients will improve with age (Sato et al., 2016; Cao et al., 2019b). In order to reduce the influence of age, we adjusted the data of height and BMD to the annual increased *Z*-score and frequency of fractures per year. Similar to the study (Trejo et al., 2017), we found that bisphosphonates can increase BMD and reduce the number of fractures, but bisphosphonates cannot improve the height of patients in our study. To have more information on the homogeneous group (type V patients), we divided patients into type V patients and non-type V patients and found that there was no significant difference between each group. It should be pointed out that due to the limitation of sample size, we did not further divide the patients into a medication group and a non-medication group, which may also be a factor affecting the results. According to Classical Sillence classification, we divided 29 OI patients in this study into types I, III, and IV, and there was no clear correlation between efficacy of bisphosphonates and phenotypes of OI.

The clinical manifestations and genetic types of OI are quite complex, and its imaging manifestations are also diverse, which is prone to missed diagnosis and misdiagnosis, especially the rare types of OI. Due to the lack of blue sclera, dentinogenesis imperfecta, and other phenotypes, it is more likely to be confused with other diseases. In this study, a total of 13 patients were misdiagnosed 32 times, and most of the diseases covered by the misdiagnosis were related to osteology, suggesting that rare types of OI patients were first diagnosed in osteology due to repeated fractures and skeletal deformity, and orthopedists should be alert to this phenomenon. If the patient has obvious scoliosis or multiple brittle fractures, especially the non-violent fractures of long bone of extremities, spine, ribs, and clavicles, it is necessary to be vigilant whether it is a rare type of OI. Most of the patients who had been admitted to our hospital had a history of repeated fractures since childhood, and scoliosis developed progressively. The spinal deformity was serious in adults, and the height was significantly lower than that of the same age. It is suggested that the patients with the above symptoms should complete the examination of biochemical markers of bone metabolism, X-ray plain film, and BMD, so as to evaluate the severity of the disease and help differential diagnosis. The serum level of calcium, phosphate, and alkaline phosphatase in OI patients is usually normal, and biochemical markers of bone turnover (including bone resorption markers and bone formation markers) are also within the corresponding normal range of children. After fracture, there may be a slight increase of biochemical markers of bone metabolism. In addition, physical examination should pay attention to whether the fontanel is closed, whether there is subluxation of radial capitulum in the upper limb, and whether there are specific facial features (such as micrognathia and midfacial hypoplasia). We suggest that genetic diagnosis should be carried out in patients with highly suspected OI in order to understand the cause of the disease and make clear the diagnosis and classification of the disease.

Although this is a retrospective study of large and rare types of OI patients, the sample size of this study is still limited, and there is also a single-center analysis that may cause some degree of bias.

## Conclusion

This study analyzed the genotype–phenotype relationship of 29 patients with rare types of OI and observed the effect of bisphosphonates. Patients with rare types of OI who received bisphosphonate treatment had an increase in lumbar spine areal BMD, a lower level of bone turnover markers, and fewer fractures. However, bisphosphonate treatment seems to have little effect on height in our study.

## Data Availability Statement

The original contributions presented in the study are included in the article/supplementary material, further inquiries can be directed to the corresponding authors.

## Ethics Statement

The studies involving human participants were reviewed and approved by the Ethics Committee of Shanghai Jiao Tong University Affiliated Sixth People’s Hospital. Written informed consent to participate in this study was provided by the participants’ legal guardian/next of kin.

## Author Contributions

Z-LZ and HZ designed the research and revised the manuscript. LX collected the clinical data, analyzed the data, and wrote the manuscript. All authors contributed to the article and approved the submitted version.

## Conflict of Interest

The authors declare that the research was conducted in the absence of any commercial or financial relationships that could be construed as a potential conflict of interest.

## Footnotes

1 http://www.le.ac.uk/ge/collagen/
